# Microglia in Neurological Diseases: A Road Map to Brain-Disease Dependent-Inflammatory Response

**DOI:** 10.3389/fncel.2018.00488

**Published:** 2018-12-18

**Authors:** Sara Bachiller, Itzia Jiménez-Ferrer, Agnes Paulus, Yiyi Yang, Maria Swanberg, Tomas Deierborg, Antonio Boza-Serrano

**Affiliations:** ^1^Experimental Neuroinflammation Laboratory, Lund University, Lund, Sweden; ^2^Translational Neurogenetics Unit, Wallenberg Neuroscience Center, Lund University, Lund, Sweden

**Keywords:** microglia, Alzheimer’s disease, Parkinson’s disease, frontotemporal dementia, regional differences, inflammation

## Abstract

Microglia represent a specialized population of macrophages-like cells in the central nervous system (CNS) considered immune sentinels that are capable of orchestrating a potent inflammatory response. Microglia are also involved in synaptic organization, trophic neuronal support during development, phagocytosis of apoptotic cells in the developing brain, myelin turnover, control of neuronal excitability, phagocytic debris removal as well as brain protection and repair. Microglial response is pathology dependent and affects to immune, metabolic. In this review, we will shed light on microglial activation depending on the disease context and the influence of factors such as aging, environment or cell-to-cell interaction.

## Introduction

Depending on the anatomical region, microglia account for 0.5–16.6% of the total cell population in the human brain (Lawson et al., 1992) and 5–12% in the mouse brain (Mittelbronn et al., 2001). Under physiological conditions, the number and function of microglia is tightly controlled by the local microenvironment and by interactions with surrounding cells. In response to an insult, microglia have the ability to shift into different functional states, modifying its proliferation (Gomez-Nicola et al., 2013), morphology (i.e., shortened processes) (Cuadros and Navascues, 1998), phagocytic activity (Sierra et al., 2013), antigen presentation (Mcgeer et al., 1988b; Jimenez-Ferrer et al., 2017) and the release of inflammatory factors such as cytokines and chemokines (Kettenmann et al., 2011). The local microenvironment and the disease conditions, where microglia are in close interaction with neurons, astrocytes and oligodendrocytes, differ between different pathologies given in the different regions. Therefore, the microglial phenotype is disease-dependent and can be regulated by biochemical and cellular composition, neuronal subpopulations and circuitries, neurotransmitter and the local metabolic rate. Aging is another factor tightly related to microglial cell activation. It has been widely studied the effect of aging in microglial cell response (Mosher and Wyss-Coray, 2014). Aging has been implicated in changes in gene expression as well as in the occurrence of dystrophic microglia, which have been linked to aberrant cytoplasmic formation, reduced ramification and fragmented processes (Streit et al., 2004). These changes related to aging, might have an impact in the progression of neurodegenerative disorders.

We will review the role of microglia in neurodegenerative diseases such as Parkinson’s disease, Alzheimer’s disease and frontotemporal dementia (FTD), where microglial activation stands as one of the key components linked to the progression of the pathology and the symptoms severity.

### Microglia Origin and Distribution

Microglial cells originate from the yolk sac and migrate into the central nervous system (CNS) during embryogenesis (Ginhoux et al., 2010). Once in the brain, they propagate and disperse in a non-heterogeneous manner throughout the CNS. In mice, a higher relative number of microglial cells is observed in the dentate gyrus of the hippocampus, the substantia nigra and parts of the basal ganglia, with the highest number of microglia present in the olfactory telencephalon (Lawson et al., 1990). Depending on the specific anatomical structure or activation profile, microglia present different morphological features (Kreutzberg, 1996; Gomez-Nicola and Perry, 2015), lysosome content (Majumdar et al., 2007), membrane composition (Button et al., 2014), electrophysiological activities (i.e., hyperpolarized resting potentials and differential membrane capacitance) (De Biase et al., 2017) and gene transcriptome profile (Chiu et al., 2013; Hickman et al., 2013).

For instance, in the basal ganglia, the expression of genes associated with a classical microglial profile such as branch dynamics and cytoskeletal regulation, inflammatory signaling, immune function and homeostasis, tend to be conserved across regions (De Biase et al., 2017). In contrast, the expression of genes involved in mitochondrial function, cell metabolism, oxidative signaling, reactive oxygen species (ROS) homeostasis and lysosomal function, are differentially expressed in different basal ganglia regions (De Biase et al., 2017).

**FIGURE 1:**
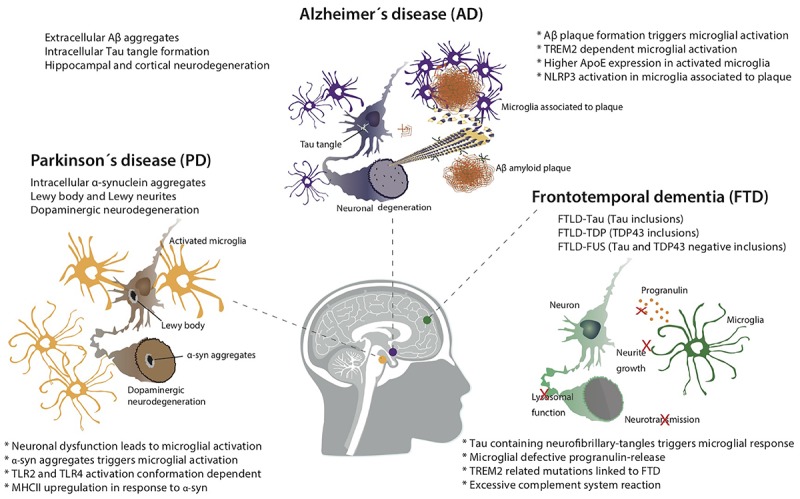
Microglial activation is involved in the progression of different neurodegenerative diseases.

Thus, a phenotypic heterogeneity of microglia can be observed within an anatomical region (Figure 1). Moreover, the transcriptomic signature affecting surface proteins and inflammatory markers can vary between microglia even in close proximity (Wu et al., 1997; Jiang-Shieh et al., 2003).

### Microglial Profile, Not M1 or M2 Anymore

Microglial phenotype regulation is mostly dependent on their interaction with molecules released by surrounding cells (neurons, microglial cells, astrocytes, etc.) through membrane-bound pattern recognition receptors (PRRs). These PRRs can be classified depending on their affinity for molecules associated to pathogens (Pathogen Associated Molecular Patterns, PAMPs) or cellular damage (Danger Associated Molecular Patterns, DAMPs) (Kawai and Akira, 2010; Kettenmann et al., 2011; Venegas and Heneka, 2017). However, not only PRRs play a role in microglial regulation. Microglial cells are also equipped with a wide variety of receptors to detect other type of molecules such as hormones and neurotransmitters (Kettenmann et al., 2011).

Traditionally, macrophage and microglial activation has been classified in two different and opposite states: classic (M1) and alternative (M2). The M1 phenotype is considered a pro-inflammatory state, in which microglial cells produce and release ROS, nitrogen reactive species (NRS) and cytokines like tumor necrosis factor-α (TNF-α), interleukin 1β (IL-1β) or interleukin 12 (IL-12). On the other hand, the M2 microglial phenotype, is considered an anti-inflammatory state and is involved in the production and release of trophic factors, such as tumor growth factor-β (TGF-β) and brain derived neurotrophic factor (BDNF) (Tang and Le, 2016). Nowadays this classification does not respond to the variety of microglial phenotype found in the brain, instead of black and white, the microglial activation profile is more a gray scale, depending on the conditions. Instead, a broad array of activation profiles and phenotypes has been recently described, especially in relation to the neurodegenerative diseases (De Biase et al., 2017; Keren-Shaul et al., 2017). For that reason, we will limit the use of the M1/M2 nomenclature all over the manuscript. For instance, Stowell et al., demonstrated a unique microglial phenotype linked to the cerebellum. Indeed, microglia are more sparsely distributed within the cerebellum, have less ramified morphology and interact dynamically with dendrites and somas of Purkinje neurons (Stowell et al., 2018). More in depth, transcriptional differences in microglial cells have been recently highlight associated to disease progression. For example, Krasemann et al., demonstrated that microglial cells play a specific role in neurodegenerative diseases depending on apolipoprotein E (APOE) and triggering receptor expressed on myeloid cells 2 (TREM2), where APOE modulation of microglial cells activation via TREM2 regulation, participate in the neuronal loss in an acute model of neurodegeneration (Krasemann et al., 2017). Indeed, APOE-TREM2 dependent microglial regulation plays a role in the regulation of homeostatic microglia. The restoration of the homeostatic microglial function prevents the neuronal loss in AD model (Krasemann et al., 2017). TREM2 has been also linked to the regulation of the microglial metabolism by sustaining cellular energetic and biosynthetic metabolism (Ulland et al., 2017), which is key to maintain the high microglial activity needed in the pathology, to deal with the excess of amyloid pathology and the plaque deposition. Further in the unique phenotype of microglial cells in the brain, it has been demonstrated that after irradiation brain-engrafting macrophages have a different phenotype compared to resident microglial cells, highlighting the importance of the microenvironment in the definition of microglial profile (Cronk et al., 2018).

Regarding expression pattern of microglial cells, De Haas et al. (2008), found throughout striatum, hippocampus, spinal cord, cerebellum, and cerebral cortex, a clear expression of CD11b, CD40, CD45, CD80, CD86, F4/80, TREM-2b, CXCR3 and CCR9, whereas no expression was found for either major histocompatibility complex II (MHCII) or CCR7.

Aging is considered the main risk factor for several neurodegenerative diseases and is accompanied by chronic altered inflammation involving changes in microglial morphology, phenotype and activity (Franceschi et al., 2007; Lopez-Otin et al., 2013). In this regard, it has been shown that region-dependent transcripts are differentially regulated during aging under physiological conditions (Grabert et al., 2016; Hanamsagar and Bilbo, 2017). Grabert et al. (2016), suggested an immunophenotypic variation of brain microglia, where cortical and striatal microglia are similar but hippocampal microglia present an intermediate profile between pro- and anti-inflammatory states.

Interestingly, most neurological conditions occur in a region-specific manner and display differential regulatory mechanisms of gene expression (Yao P. et al., 2015). Another factor to be considered is the relation between gray and white matter and microglial activity. For instance, trauma-induced lesions the spinal white matter exhibited a greater microgliosis than spinal gray matter. So, one can speculate with myelin composition as a factor that influences the microglial activity (Batchelor et al., 2008). In fact, greater inflammatory response was found in white matter compared to gray matter within both the brain and spinal cord (Batchelor et al., 2008).

### Regional Differences: Hippocampus, Frontal Cortex and Substantia Nigra and Striatum

#### Hippocampus

Hippocampus is an essential learning and memory structure located bilaterally, deep in the temporal lobes of the gray matter (Dhikav and Anand, 2012). Hippocampus plays a critical role in learning and memory processes (Shen et al., 1994; Mu and Gage, 2011) and regional measures of hippocampal atrophy, are one of the strongest predictors of Alzheimer’s disease (AD) progression (Henneman et al., 2009). Moreover, microglial activation in the hippocampus has been study and recently, Grabert et al., described how microglial cells in the hippocampus decrease the expression of genes related to environment sensing (Grabert et al., 2016), making the hippocampus more vulnerable to aging and disease related protein deposition. They also demonstrated that in healthy conditions, microglial cells in the hippocampus present a higher “immune-vigilant” phenotype. This can be linked with a higher microglial response in AD pathology to plaque formation, giving rise to a harmful chronic inflammatory response. Another particular features of the hippocampus that might influence microglial activity, are the gray matter content and the microglial density (Lawson et al., 1990). For instance, Hart et al., demonstrated a significant regional difference in microglial phenotypes, with the microglia of white matter exhibiting greater up regulation of CD11b, CD68, CD11c, F4/80 and FcγRI than gray matter (Hart et al., 2012). In another study by De Haas et al. (2008), microglia from hippocampus displayed the lowest expression for four of the proteins (CD45, CD80, CXCR3, and CCR9) and the highest expression for F4/80. CXCR3 receptor has been shown to be involved in neuron-microglia communication, so that, lower values of CXCR3 might impair the communication, resulting in less microglial control. According to Krauthausen et al. (2014), the CXCR3 chemokine system is critically involved in the intrinsic glial activation during demyelination, which significantly modulates the distribution of glial cells and the local cytokine milieu. In another study, Rappert et al. (2004), found CXCR3 essential for microglial recruitment and neuronal reorganization.

Aging, is also affecting neuronal network in the hippocampus. For instance, older adults had poorer connectivity between with the posterior brain regions than young adults. On the contrary, older adults showed increased connectivity between the hippocampus and frontal regions (Dennis et al., 2008). Differences in neuronal network might implicate changes in microglial activity pattern.

All the factors before mentioned might predispose microglial activity for hippocampal-related brain diseases such as Alzheimer’s disease, where microglial activation and neuroinflammation play a key role (Dansokho and Heneka, 2017).

#### Frontal Cortex

Frontal lobes, including prefrontal cortex (PFC) and motor areas, encompass around 30% of the cortical surface of the human brain (Zinn, 2008; Kurz et al., 2014). They are involved in the execution of high-order tasks such as planning, executive function, working memory and in initiate and direct physical movement (Macht, 2016). Temporal lobes represent the latero-basal part of the cortex and they have been related with receptive and expressive language processing, semantic memory and social concepts (Wong and Gallate, 2012). Both areas are widely connected, been critical to language processing but also to the top-down behavioral control (Brass et al., 2005). In 1997, Raz et al., performed a cross-sectional study of the cortex using structural neuroimaging. The study indicates that PFC shows the highest degree of age-related atrophy. Indeed, the most substantial age-related decline was found in the volume of the prefrontal gray matter, compared to another areas such as inferior temporal and superior parietal cortices (Raz et al., 1997).

Regarding microglial phenotype in healthy cortex, CD11b on microglia from cerebral cortex was repeatedly lower than from spinal cord, and CD40 was lower in cortex compared to cerebellum. On the other hand, CXCR3 was higher in the cortex compared to the cerebellum (De Haas et al., 2008). As previously mentioned, CXCR3 has been linked to glial accumulation and activation in culprizone-demyelination model (Krauthausen et al., 2014).

#### Substantia Nigra and Striatum

The substantia nigra is a gray matter area caudal to the hippocampus with a particularly high microglial density (Lawson et al., 1990), which is part of the basal ganglia and it is located in the midbrain. Its name is due to the high levels of neuromelanin contained in the dopaminergic neurons (Zecca et al., 2003). Aging is a key factor in the survival of dopaminergic neurons in the substantia nigra, experiments a decrease in the number and size of pigmented neurons in the substantia nigra pars compacta (SNpc) over time (Ma et al., 1999).

In this structure, microglial cells present a proportion of 12%, which is a particular dense population (Lawson et al., 1990). A key receptor to control microglial activity is fractalkine receptor (Sheridan and Murphy, 2013). Notably, the fractalkine receptor distribution is higher in the striatum than in other structures, such as cerebellum (Tarozzo et al., 2003). Another important receptor involved in microglia-neuron interaction is CD200, which is also affected by aging in the substantia nigra. In fact, Wang et al. (2011), found CD200 downregulated in age dependent-manner in wild-type mice.

The number of microglial cells in the substantia nigra, the differential regulation of fractalkine and CD200 and the decrease of dopaminergic neurons are environmental factors that might modulate microglial activity. For instance, high microglial density, the reduction of CD200 receptor, or the decrease in dopaminergic population, can decrease the regulation of microglial cell by neurons. An environment with less microglial regulation, or overpopulated, might give rise to a reduction of trophic factors production or excessive inflammatory molecules production, which can be detrimental in the long run.

## Alzheimer’s Disease Pathology

Although AD is the most common form of dementia, caused by neuronal death (Santiago et al., 2017), its pathology and underlying causes are not fully understood. AD is associated to extracellular deposition of amyloid-β (Aβ) plaques due to an increased production and/or lack of clearance of Aβ peptides derived from amyloid precursor protein (APP) cleavage (Mawuenyega et al., 2010; Sarlus and Heneka, 2017) and by abnormal intraneuronal accumulation of hyperphosphorylated tau protein (Johnson and Stoothoff, 2004) (Figure 1). In normal conditions, tau stabilizes microtubules but in pathological conditions, intracellular accumulation of hyperphosphorylated tau disorganizes microtubules impairing the cytoskeleton. This has been linked to neurodegeneration and cognitive impairment (Ossenkoppele et al., 2016; Bejanin et al., 2017).

Aging is a primary risk factor for AD (Kawas and Corrada, 2006; Guerreiro and Bras, 2015) and AD can be genetically classified into familial or idiopathic forms. The early-onset form of AD (younger than 65 years old) is predominantly familial and most often linked to single mutations in the genes encoding APP, presenilin 1 (PSEN1) or presenilin 2 (PSEN2). However, around 90% of the patients display an idiopathic form of AD, which is considered a multifactorial disease, caused by an interplay between environment, genetics and lifestyle (Winblad et al., 2016). Further, idiopathic late-onset AD (older than 65 years old) is associated to a strong activation of the innate immune system, pointing out neuroinflammation as a strong contributor to the pathogenesis of AD (Heneka et al., 2015). Genome-Wide Association Studies (GWAS) have identified over 20 genetic loci that are robustly associated with AD risk and microglial immune responses (Lambert et al., 2013; Karch et al., 2014).

### Microglial Activation in Alzheimer’s Disease

It has been proposed that both functional and morphological changes of the hippocampus are associated with changes in microglial cell response during AD progression (Henneman et al., 2009).

As previously mentioned, PRRs bind to different DAMPs or PAMPs. For instance, Aβ species can trigger an inflammatory response in microglial cells (Venegas and Heneka, 2017). Amyloid-like structures are expressed by microorganisms such as bacteria (Kreutzberg, 1995), being microglial cells the first line of defense of the innate immune system in the brain. This fact would provide a potential evolutionary explanation for the microglial response against Aβ. In this section, we will discuss the role in AD pathology for the main PRRs-associated risk factors, including, ApoE, TREM2, CD33, toll-like receptors (TLRs) and inflammasome.

The ApoE is a protein involved in the cell metabolism regulation and transportation of fatty acids. ApoE can be found in four different alleles. For instance, ApoE 4 allele increases the risk of AD threefold to eightfold, depending on the number of allele copies. ApoE has three common variants: ApoE 2, which is rare and may protect against AD; ApoE 3, which is common and seems to play a neutral role; and ApoE 4, which is a major risk allele for AD (Kim et al., 2009; Iaccarino et al., 2018).

Human ApoE has also been demonstrated to play a role in tau pathology and brain inflammation^53^. Shi et al. (2017), generated tau transgenic mice on ApoE 2, ApoE 3 or ApoE 4 knock-in and also ApoE knockout background. The results demonstrated that ApoE4/tau transgenic mice have higher tau levels leading to greater rates of disease progression and neuroinflammatory responses, while absence of ApoE presents a protective effect. It indicates that *APOE* levels could influence tau pathology and tau-mediated neurodegeneration independently of amyloid-β. As a future approach, it would be interesting to target ApoE4 in order to reduce tau pathology in neurodegenerative diseases.

Innate immune system has been proved to be strongly involved in AD pathogenesis. Indeed, genetic findings provide strong evidence for a pivotal role of microglial activation in AD pathogenesis. These include a common variant of Transcription factor PU.1 (SPI1) for microglial development and function (Kierdorf et al., 2013; London et al., 2013), which is associated with a reduced risk of AD (Huang et al., 2017), as well as an allelic variant of the TREM2, a cell-surface receptor exclusively of microglial cells (Colonna, 2003) which is associated to increased risk for developing AD up to threefold (Jonsson et al., 2013; Abduljaleel et al., 2014; Ulrich et al., 2017). TREM2 encodes a type I transmembrane glycoprotein of 40 kDa, that contains an extracellular immunoglobulin domain (Raha-Chowdhury et al., 2018). This protein is expressed on myeloid cells such as tissue macrophages, dendritic cells and microglia (Hickman and El Khoury, 2014).

Under normal conditions, TREM2 promotes phagocytosis, proliferation and survival. However, AD risk variants of TREM2 (e.g., TREM2 R47H, homozygous mutations or deletions, and heterozygous expression of TREM2 variants) impair the proper function of microglia in terms of phagocytosis, inflammatory response, energy metabolism, plaque compaction and activation, affecting disease progression (Ulland et al., 2017; Yin et al., 2017; Zhong et al., 2017). Studies in TREM2-deficient mouse models are giving conflicting results on AD pathology (Jay et al., 2017). For instance, TREM2-deficient APP-PS1 AD mice displayed reduced accumulation of microglia around plaques and a decreased inflammatory response but did not show any differences in amyloid burden (Ulrich et al., 2014; Wang et al., 2015). However, Jay et al. (2017), reported that the TREM2 response could be age-dependent, resulting in decreased number of plaques at early age (4 months) and increasing number of plaques in late stages of the pathology (8 months) (Wang et al., 2015). Further, TREM2 studies in transgenic mice of tau pathology also described conflicting results in two different animal models. *Trem2* deletion seems to play a more neuroprotective role in the PS19 tau model (Leyns et al., 2017) but becoming harmful in the hTau model (Bemiller et al., 2017). In regards to TREM2 and ApoE, Krasemann et al. (2017), describe an ApoE- and TREM2-dependent microglial response that was linked to neurodegenerative-associated phenotype (MGnD) in brain tissue. They suggest that the transition from homeostatic to MGnD microglia is ApoE-dependent in aged mice in mouse model of AD. The homeostatic profile of microglial cells is characterized by low APOE expression and controlled by TGF-β signaling, while the MGnD microglial phenotype is characterized by activation of TREM2 signaling and a higher APOE expression. MGnD microglial profile is also triggered by cellular debris accumulation in aged mice or in neurodegenerative diseases. Interestingly, in *APOE* or *Trem2* knockout mice, microglial response becomes attenuated, suggesting that blocking the transition could be a possible route for therapeutic intervention in AD.

Another interesting gene linked to AD is the microglial chemokine receptor (Cx3cr1) involved in microglial migration and in neuron/microglial activity regulation (Sheridan and Murphy, 2013). In tau transgenic mice, deficiency in Cx3cr1 leads to activation of microglial cells and tau pathology progression in terms of protein aggregation (Maphis et al., 2015). In alternative study, Cx3cr1 knockout AD mouse model showed a prevent neuronal loss but fails in alter amyloid burden (Fuhrmann et al., 2010).

Additional gene considered a risk for AD pathology, is CD33. CD33 is a transmembrane receptor mainly expressed by microglial cells in the brain that regulates innate immune response. Increased levels of this receptor have been correlated with an elevated risk of AD due to slower microglial phagocytosis and Aβ clearance, leading to an increased in plaque deposition in AD mice brains (Griciuc et al., 2013; Jiang et al., 2014).

Additionally, PRR family TLRs has being linked to AD. TLR are a subfamily of PRRs, where most of them are expressed by microglia. TLRs-mediated signaling has been suggested to be both detrimental and beneficial depending on the receptor involved in the response (J Downer, 2012). Thus, toll-like receptor 9 (TLR9) elicits an innate-immune response and clearance of Aβ through activation by unmethylated cytosine-guanosine (CpG) reducing AD pathology in mice (Scholtzova et al., 2014). However, microglial activation can play a detrimental role, depending on the TLR activated. For instance, toll-like receptor 4 (TLR4), expressed in microglia, plays an important role in neuroinflammation (Zhang et al., 2015) and can be stimulated by both fibrillar and oligomeric forms of Aβ (Dansokho and Heneka, 2017). However, TLR4 has also been suggested be protective in AD (Minoretti et al., 2006; Michaud et al., 2013), that could be depend on other parameters such as activation intensity. Further, activation of another TLR—toll-like receptor 2 (TLR2), which can be also stimulated by fibrillar Aβ, has a detrimental impact on microglia activating the cells into a more pro-inflammatory profile (Dansokho and Heneka, 2017). Each TLR is stimulated by different ligands, especially TLR2 and TLR4 (Landreth and Reed-Geaghan, 2009; Wang et al., 2016), however, both receptors are stimulated by Aβ, and it has been described that they form of complexes together with their co-receptors (Mcmanus and Heneka, 2017), CD14 and CD36, initiating an intracellular signal cascade, leading to the expression of the pro-inflammatory molecules. For instance, CD14 is associated with the TLR2-TLR4 dimerization and complex with the ability to recognizes and bind Aβ. In fact, CD14, TLR2 and TLR4 deficiency leads to a reduced microglia phagocytic activation and production of ROS (Landreth and Reed-Geaghan, 2009). On the other hand, CD36 regulates TLR4-TLR6 heterodimer-depending inflammatory response to Aβ, being involved in inflammasome activation in AD (Stewart et al., 2010). Inflammasome consists in a large protein complex, being nod like receptor protein 3 (NLRP3) one of the most well characterized-inflammasome-related proteins in AD (Sheedy et al., 2013). NLRP3 forms a complex with caspase-1 and ASC by their effector domains. Once the complex is activated, it initiates cleavage of pro-IL-1b and pro-IL-18 into IL-1b and IL-18 (Latz et al., 2013), both inflammatory cytokines (Heneka et al., 2013; Guo et al., 2015; Karve et al., 2016; Mcmanus and Heneka, 2017). It has been suggested that NLPR3 activation is taking place by cathepsin B release in the cytoplasm, triggering the formation of the NLRP3 inflammasome complex (Bai et al., 2018). In fact, accumulation of cathepsin B in microglial cells has been described around senile plaques and in AD mice models and its inhibition, decreases the amyloid plaques as well as improves memory deficits (Mueller-Steiner et al., 2006; Hook et al., 2008).

Another major mediator in inflammation is the complement system, which evolved to protect the host against infection and plays an important role in microglia-mediated synaptic refinement during brain development. The complement system is involved in the innate immune response and its main role is clearing cellular debris, damage and apoptotic cells (Carroll and Isenman, 2012) by enhancing the antibody’s activity; however, its activation can even lead to early synapse loss in AD context (Hong et al., 2016a). Thus, studies of complement proteins are an interesting approach to investigate neurodegenerative diseases like AD. A gene variant of the complement component receptor 1 (CR1-B/S) has been identified as one of the most important risk genes for late-onset Alzheimer’s disease (LOAD) (Crehan et al., 2012). In addition, the complement component 3 (C3), which plays a central role in the complement system, has been shown to be upregulated in AD, increasing microglial phagocytic capacity (Lian et al., 2016). Interestingly, a recent study done by Shi et al. (2017), showed that in APP/PS1 AD mouse model, lack of C3 is actually protecting from both synapse loss and cognitive decline, probably due to an altered response of glial cells. Thus, modulation of complement signaling may have potential as a new therapeutic strategy for Alzheimer’s disease.

Microglia is also involved in synapses elimination, mainly those with less activity or from dysfunctional neurons, by a process called synaptic pruning (York et al., 2017). Further, microglia seem to drive synapse loss in neurodegenerative diseases (Presumey et al., 2017). Indeed, abnormal synapse loss in AD also correlates with cognitive decline (Bahrini et al., 2015; Hong et al., 2016a). However, even if first amyloid plaques appear years before clinical symptoms of AD, tau pathology correlates better with cognitive impairment in AD (Jack et al., 2010).

Another key inflammatory molecule involved in AD is the nitric oxide synthase (NOS) (Kummer et al., 2011). The production of the inducible form (iNOS) is highly linked to microglial activation, being one of the key molecules in the inflammatory response. Indeed, Kummer et al. (2011), demonstrated a key role for nitric oxide related to a post-translational modification of the Tyrosine in the position number 10 of the APP sequences that confers a higher aggregation capacity. This fact makes iNOS a potential therapeutic target in order to avoid the aggregation of the proteins and the plaque deposition in AD.

Cytokines such as IL-12 have been linked to AD progression. For instance, the lack of IL-23 reduces AD pathology in APP/PS1 model (Vom Berg et al., 2012), affecting the total brain amyloid burden in mice lacking of IL-12.

To summarize, innate immune activation and inflammatory response driven by microglial cells are currently rising up as one of the key research areas to understand AD pathology progression, as well as, to find new therapeutic targets to halt the progression of the pathology.

## Frontotemporal Dementia

Chronic or progressive loss of cortical and subcortical functions have been defined in FTD (Gotz et al., 2006). This disorder is the third most common form of dementia, after AD and dementia with Lewy bodies (DLB), and is a leading type of early-onset dementia (Vieira et al., 2013). Frontotemporal lobar dementia (FTLD) is a neurodegenerative disorder characterized by neuronal loss, gliosis and microvascular changes of frontal and temporal lobes, resulting in cognitive decline, accompanied by mood, behavior and personality disturbances (Gotz et al., 2006; Ridolfi et al., 2013; Bang et al., 2015; Zhang, 2015) (Figure 1). FTLD can be subdivided in three different types, attending to the main protein component of neuronal and glia abnormal deposition and their distribution (Broe et al., 2003; Mackenzie et al., 2010; Bahia et al., 2013): FTLD-Tau (related to microtubule-associated protein Tau) is the most common subtype, reaching 50% of prevalence (Hutton et al., 1998; Dickson et al., 2011); FTLD-TDP (associated to the transactive response DNA-binding protein 43 kDa) presents slightly less prevalence, around 45% (Bigio, 2011); and FTLD-FUS (related to fused in sarcoma protein, FUS), appears only in ∼5% of the cases (Weintraub and Mesulam, 2009).

Cortical atrophy and synaptic loss have been also described in AD, the most common type of dementia (Morrison and Hof, 2002; Ridolfi et al., 2013; Hong et al., 2016b). AD is characterized by amyloid-beta-containing plaques and tau-containing neurofibrillary tangles whereas FTLD mainly exhibits tau-containing neurofibrillary tangles (Gotz et al., 2006), despite of the presence of amyloid beta (Tan et al., 2017). This abnormal protein accumulation triggers the innate immune system in the brain (Bellucci et al., 2011; Ridolfi et al., 2013).

In the last few years, several studies show that chronic microglial activation participates in the development and/or progression of neurodegenerative disorders, such as AD and FTLD [for review see (Ridolfi et al., 2013; Pasqualetti et al., 2015)].

### Microglial Activation in Frontotemporal Dementia

In the last years, several studies have shown that alterations in protein aggregation and neuroinflammation may begin at early stages of FTLD or AD (Yoshiyama et al., 2007; Heneka et al., 2014; Morales et al., 2014). In fact, *in vivo* PET imaging studies, showed an increased binding of 11-C-R-PK1195 microglial ligand in frontotemporal brain regions, suggesting that microglial activation could start at early stages of FTLD prior to anatomical changes (Rosso et al., 2003; Cagnin et al., 2004). Similar results were also found in AD patients where, in addition, activated microglia correlates with cognitive decline (Edison et al., 2008).

FTLD and AD show microglial activation pattern that reflects the distribution of the pathology of both diseases. In this way, a higher microglial activation in the frontal subcortical white matter in FTLD patients has been reported, whereas this activation is more prominent in temporal regions in AD patients (Lant et al., 2014; Taipa et al., 2017).

Regarding to molecular level, some genes related with microglial activation have been associated with FTLD and AD (Guerreiro et al., 2013; Lue et al., 2015; Lui et al., 2016). One of the most well reported is Progranulin (*PGRN*) (Heneka et al., 2014). This protein is constitutively expressed and secreted by microglia and participates in cell-cycle regulation, wound repair, axonal growth and as a mediator of the inflammatory response (Toh et al., 2011; Mao et al., 2017). PGRN protein deficiencies have been related with a progressive upregulation of lysosomal and innate immune genes in FTLD (Lui et al., 2016). Furthermore, it has also been described that mutations in PGRN induce a higher microglial activation, characterized by reactive morphology, and excessive complement system reaction in the frontal cortex of FTLD patients, leading to defective synaptic pruning and inducing neuronal dysfunction (Lui et al., 2016). In FTLD patients, microglial activation and HMGB1 (High mobility group box 1) have been found co-localizing with tau oligomers, which might indicate the initiation of an inflammatory response by microglial cells. This inflammatory response might actively participate in the neuronal loss due to the creation of a toxic environment (Nilson et al., 2017).

In recent years, TREM2, another gene related to microglial function, is attracting the attention of neuroscientists due to its dual role in FTLD and AD. This gene is expressed by microglial cells in brain in the cortical areas, as well as hippocampus (Zhang et al., 2014; Yeh et al., 2017). Moreover, up-regulation of TREM2 protein has been linked to FTLD cases. *TREM2*-related mutations (c.40 + 3delAGG, Q33X, Y38C, T66M, D86V, W198X) (Chouery et al., 2008; Giraldo et al., 2013; Guerreiro et al., 2013) have been found in individuals from six different families, presenting different forms of frontotemporal dementia [further, see review (Yeh et al., 2017)]. As well as amyloid, tau tangles accumulated within neurons in the temporal lobe of FTLD patients induce microglial activation, leading to TREM2 expression.

To summarize, some evidences suggest that neuroinflammation could be a common process associated with neuronal damage and cognitive impairment, symptoms that dementia patient’s exhibit. A better understanding of neuronal-glial (especially microglial and astrocytes) mechanisms involved in the cortical degeneration is a current challenge to identify specific targets that may prevent the development and/or the progression of cortical-related neuronal disorders.

## Parkinson’s Disease

Parkinson’s disease (PD) is a motor brain disorder characterized by motor impairment due to degeneration of dopaminergic neurons. Innate immune response in PD has been widely studied and compelling evidences demonstrated the role of neuroinflammatory response in the progression of PD (Perry, 2012). Other structure highly relevant in PD pathology is the Locus Coeruleus (LC), which we will explain more in detail later.

The main pathological feature of PD is the death of dopaminergic neurons in the SNpc (Brettschneider et al., 2015). These neurons project their axons from the SNpc to the striatum. The loss of these neuronal connections is the main cause of motor impairment suffered by PD patients. However, the loss of dopaminergic neurons occurs in many other regions such as: LC, dorsal motor of the vagus nerve, amygdala or hypothalamus (Dickson, 2012). The aggregation of misfolded α-synuclein in intraneuronal inclusions, called Lewy bodies, is another significant feature of the pathology. These inclusions can be found in neuronal somas [Lewy’s bodies (LB)] or in the neurites (Lewy’s neurites) (Spillantini et al., 1998) (Figure 1). Although α-synuclein inclusion is the main pathological hallmark of PD, they can be found in other pathologies such as DLB (Mckeith, 2004).

Another important aspect of the biology of PD is the role of α-synuclein in the pathogenesis of the disease. It is known that α-synuclein can be present in different aggregation states, from monomers to small fibrils, being the main compound of the Lewy bodies. For instance, oligomers of α-synuclein are toxic for neurons (Ingelsson, 2016).

From the clinical point of view, we can explain the progression of the disease according to Braak’s hypothesis, based on the spread of α-synuclein pathology and its effects over the time (Brettschneider et al., 2015). The production of α-synuclein, its deposition and spreading within neurons, would induce their malfunctions leading to neuronal dysfunction and eventually neuronal death. However, only in advanced stages of PD, intraneuronal α-synuclein aggregation causes the loss of dopaminergic neurons in the SNpc (Braak et al., 2003). Being like this, we might think that factors other than protein aggregation might play a role in the neuronal death, for instance, the activation of the innate immune system. Indeed, cell death induces the release of different molecules, including α-synuclein and inflammatory factors. This microglial activation leads to an inflammatory response, which may play an important role in the progression of the pathology (Perry, 2012).

Another important structure involved in PD is the LC, which is a small brainstem nucleus located on the rostral side of the fourth ventricle in the CNS. It is also a major source of norepinephrine (NE) in CNS. NE was one of the first neurotransmitters to be identified by Swedish physiologist Ulf von Euler in 1945 (Euler, 1945). Besides its role in the periphery, it is an essentially modulatory neurotransmitter of the CNS (Dahlstroem and Fuxe, 1964). Essentially, the locus coeruleus-norepinephrine (LC-NE) system is involved in regulation of several behaviors, including sleep-waking cycle, alertness, learning and memory as well as stress response (Berridge and Waterhouse, 2003). Moreover, early symptoms of PD, like sleep disorder, depression and autonomic nervous dysfunction, which are developed before emergence of motor impairment, are related to LC neuronal degeneration (Espay et al., 2014).

The integrity of LC and the connectivity between this structure and the rest of the brain, are crucial to regulate a variety of responses throughout the CNS. Impairment of LC function has been suggested to contribute to many neurodegenerative diseases, such PD (Schwarz and Luo, 2015). Besides the neuronal loss, reduction of NE could aggravates neurodegeneration due to its role as a potent anti-inflammatory and neuroprotective mediator (Feinstein et al., 2016). So that, the role of the innate immune response seems to be essential to understand how works the PD in the LC.

### Microglial Activation in Parkinson’s Disease

Aside from the degeneration of dopaminergic neurons in the SNpc and intraneuronal synuclein deposits, inflammation is a key component in PD. The concentration of microglia is particularly high in SNpc. The inflammatory response in PD is mainly carried out by microglial cells and to a minor extent by astrocytes (Perry, 2012). As we mentioned in the introduction, microglial cells are associated to the clearance of neuronal death-related debris. Indeed, this death cell related debris can activate microglia, leading to the release of different inflammatory factors, such as, pro-inflammatory cytokines, chemokines, etc., along with ROS and reactive nitrogen species (RNS). Thus, neuroinflammatory responses may contribute to the progression of the pathology.

Microglial activation in PD has been extensively documented. In 1988, one study by Mcgeer et al. (1988a) showed human leukocyte antigen (HLA) receptor reactivity in the SNpc of PD patients. Apart from microglial activation, different neuroinflammatory factors have been found in the brain of PD patients (Qian et al., 2011). Elevated levels of TNF-α, IL-1β, IL-6 and Interferon-γ (IFN-γ) have been detected in striatum and substantia nigra of PD patients compared to healthy controls (Mogi et al., 1994a,b; Hunot et al., 1999; Sawada et al., 2006). Another pro-inflammatory molecules, NRS, has been measured in glial cells of PD patients in the vicinity of dopaminergic neurons in the brain (Hunot et al., 1996). The production of nitric oxide by glial cells might be toxic for dopaminergic neurons (Boje and Arora, 1992). In fact, the *in vitro* inhibition of NRS protects neurons in co-culture with lipopolysaccharide (LPS) activated microglial cells (Boje and Arora, 1992). Moreover, different pro-inflammatory cytokines production might induce a toxic effect in neurons expressing their receptor. For example, TNF-α is highly expressed in dopaminergic neurons of PD patients, which might exert a deleterious effect in an inflammatory context (Mogi et al., 2000).

One of the most important factors linked to microglial activation in PD is the MHCII. Microglial MHCII expression has been mainly associated to neurons containing Lewy bodies, damage neurons and neurites in PD brains (Imamura et al., 2003). Moreover, the cells expressing MHCII were also positive for pro-inflammatory markers such us IL-6 or TNF-α. In another study, Harms et al. (2013), showed microglial cells close to AAV2-synuclein-positive neurons expressing MHCII. The expression of MHCII was increased in a time dependent-fashion. Moreover, MHCII knockout mice showed less neuronal loss (Harms et al., 2013). GWAS studies in PD patients have also highlight the relation of MHCII gene variants with the etiology of the disease, being MHCII-related SNP considered a risk factor (Pierce and Coetzee, 2017).

Moreover, there are several known inflammatory-associated risk factors that might increase the probability of developing PD. For example, TNF-α polymorphism at position 308, IL-6 polymorphism at position 174 and IL-1β at position 511, are more common in PD patients than in healthy controls (Kruger et al., 2000; Hakansson et al., 2005; Arman et al., 2010). Still, it is not fully clear in which way these polymorphisms affect the progression of PD pathology, whether the mutations increase the basal levels of pro-inflammatory factors or exacerbate the inflammatory response upon insult.

In order to resemble the main pathological hallmarks of the PD, several animal models have been developed and most of them present microglial activation and inflammatory response along with the progression of the pathology. Up to date, toxic-based models such as, 1-methyl-4-phenyl-1,2,3,6-tetrahydropyridine (MPTP), 6-hydroxidopamine (6-OHDA) and LPS are the main PD mice models used, due to their ability to induce cell death and microglial activation (Castano et al., 1998; Barcia et al., 2004; Rodriguez-Pallares et al., 2007). The link between the inflammatory response and the neuronal death has been assessed in different studies, giving a positive correlation between both factors. For example, the MPTP models provide robust neuronal death and microglial activation in the SNpc (Barcia et al., 2004; Drouin-Ouellet et al., 2011; Noelker et al., 2013). In fact, iNOS knockout mice show a reduction of neuronal death upon MPTP injections compared to WT mice (Liberatore et al., 1999).

Parkinson’s disease mouse model based on LPS injections, is able to selectively induce dopaminergic cell death in the SNpc more efficiently than in the striatum (Herrera et al., 2000). Removing microglial cells have been successfully tested in animal models. For instance, Venero et al., injected minocycline, a compound used to inhibit microglial cells, in an animal model of intranigral injection of LPS. As a result of the microglial depletion by minocycline, the levels of TNF-α, IL-1α and protein peroxynitration, were reduced and the dopaminergic neurons were protected compared to minocycline non-injected mice (Tomas-Camardiel et al., 2004).

Moreover, *in vivo* models of α-synuclein overexpression showed microglial activation. In fact, overexpression of a mutant form of α-synuclein (A53T and A30P) induces microglial activation in a transgenic mouse model leading to early pro-inflammatory microglial activity (Su et al., 2009). Upon LPS injection, mice expressing a mutant version of α-synuclein (A53T) present selective dopaminergic cell death in the SNpc along with formation of α-synuclein aggregates within neurons, a classical hallmark of PD pathology. Interestingly, inclusions contained α-synuclein nitrated residues, which can be linked with the inflammatory response elicited upon LPS injection (Gao et al., 2008). Additionally, *in vitro* models consisting of α-synuclein overexpression display the classical features of microglial activation, as well as the release of different pro-inflammatory factors (Su et al., 2008).

Regarding PRRs, such as TLR4 and TLR2, have been demonstrated that are able to bind to α-synuclein in multiple system atrophy (MSA) models, where the overexpression of α-synuclein increases motor impairment as well as dopaminergic degeneration in the SNpc. Furthermore, α-synuclein binding to TLR4 enhances microglial phagocytic capacity as well as the production of pro-inflammatory factors, such as TNF-α (Stefanova et al., 2011). Indeed, the lack of TLR4 reduces microglial α-synuclein-dependent activation, reducing the production of inflammatory species (Fellner et al., 2013). The important role of TLR4 in the inflammatory response was also evaluated using MPTP as an inducer of Parkinson-like lesion in mouse. In fact, the lack of TLR4 reduced the inflammatory response, preventing the dopaminergic cell death, which is linked to reduced microglial activation (Noelker et al., 2013). Moreover, neuron-released α-synuclein can induce an innate immune response, activating microglial cells by TLR2 (Kim et al., 2013). Along with TLR2, TLR1, has been also linked to microglial response in PD. Indeed, TLR1/TLR2 heterodimer plays a critical role in α-synuclein response. TLR1/TLR2 heterodimer acts through MyD88 adaptor activating nuclear factor κ B (NFkB) pathway, which leads to pro-inflammatory cytokine production (Daniele et al., 2015).

The main pathways involved in the inflammatory process in PD are very similar to the one elicited in AD. C-Jun N-terminal Kinase (JNK), mitogen-activated protein kinase (MAPK) or nuclear factor κβ (NFκβ) pathways are mainly involved in the inflammatory response in PD. For instance, MPTP mouse models trigger the activation of cyclooxygenase 2 (COX2) by JNK2 and JNK3 related to JNK pathways. Indeed, JNK2 and JNK3 deficiency reduces dopaminergic cell death (Hunot et al., 2004). The activation of MAPK p38 pathways in MPTP models has also been shown in dopaminergic neurons in the SNpc (Karunakaran et al., 2008). Finally, NFκβ pathway has been found upregulated in microglia and astrocytes of PD patients intoxicated with MPTP. NFκβ inhibition reduces microglial activation and mRNA levels of TNF-α, IL-1β and iNOS in the SNpc, protecting the neurons located in the nigra-striatum pathway and improving motor ability in mice exposed to MPTP (Ghosh et al., 2007).

Altogether, the microglial activation and the consequent inflammatory response taken place in PD is a major component of the pathology, having a key role in the progression of the disease. However, the exact mechanism governing the activation of microglial cells and how is that affecting to the neuronal survival still remains unclear.

#### Microglial Activation in Parkinson’s Disease: The Role of Locus Cereleus

Parkinson’s disease is commonly characterized with specific loss of dopaminergic neurons in SNpc and also presence of LB in many of the remaining neurons. However, compelling evidence has shown that degeneration of NE neurons within the LC is more significant and earlier than that in SNpc (Fornai et al., 2007; Espay et al., 2014). Using mouse models of PD, Yao et al(2015a) have found depletion of NE in the LC promotes dopaminergic neuron degeneration by modulating microglial activity in the midbrain, which lead to increased expression of pro-inflammatory cytokines, diminished neurotrophic factors and damaged ability of anti-oxidation in the SNpc (Yao N. et al., 2015). In addition, microglia can express β-adrenergic receptors in response to NE, which mediates the suppression of pro-inflammatory molecules, such as TNF-α, IL-1β and iNOS, as well as enhancement of anti-inflammatory molecules production (Feinstein et al., 2002). Thus, deficient production of NE suppresses the anti-inflammatory function on microglia in CNS. So that, the reduction of LC noradrenergic neurons also occurs prior to neuronal degeneration in other brain areas, which has been linked to microglia activity dysregulation (Feinstein et al., 2002). Furthermore, NE has also been related to changes in the mobility of microglia, impairing its capacity to detect and respond to damage tissue *in vivo* through adrenergic receptors (Gyoneva and Traynelis, 2013).

Taken together, LC-NE system has broad actions in the nervous system and provides neuroprotective actions by modulation of microglia activity in regard to inflammation, trophic factors release or motility. So that, therapeutic methods aiming to increase NE levels have the potential to slow down the progression of neurodegeneration by suppression of inflammation. However, so far there is no effective ways available to test this approach in patients. Although many studies have been done over past years to understand LC-NE system, we still need improve our understanding of these disorders in future.

## Future Directions

Compelling evidences shed light on the role of innate immune system and microglia response in the progression of neurodegenerative diseases, such as PD or AD. Microglial cells can develop different functional activation states, actively participating in brain homeostasis. However, an uncontrolled inflammatory response by microglial cells can lead to a detrimental outcome. Mainly, JAK/STATs and NFkB pathways govern microglial pro-inflammatory response and molecules such as TNF-α, IL-1β and iNOS are the main outcome the downstream signaling of these pathways. This activation is linked to a deleterious role in neurodegenerative diseases, expanding the inflammatory response and increasing the neuronal damage.

Being like that, to actively counteract the progression of the pathology by regulation of microglial pro-inflammatory activation, is a promising therapeutic target. So far, efforts have been made on different molecular targets. For instance, capsaicin has been used to block TNF-α or IL-1β, reducing the neuronal degeneration induced by MPTP in PD models (Chung et al., 2017). Similar to TNF-α or IL-1β, capsaicin significantly suppressed the activation of MAPK related pathways. The mechanism seems to act through Prostaglandin E2 (PGE2) pathways (Bhatia et al., 2017). Another promising target to prevent microglial-related inflammatory response is COX2. Recently, COX2 has been shown that plays a key role in 6-hydroxydopamine (6-OHDA) by increasing the levels of PGE2. Being like that, to inhibit COX2 would be an interesting approach to impair microglial inflammatory response. Indeed, the suppression of this protein has been linked with improved memory outcome in Tg2576 AD mouse models (Kotilinek et al., 2008). This pathway seems to work through PGE2 rather than reduction of inflammatory response. Other inflammatory pathways, such as NLRP3 inflammasome or TLR4 pathways, became a therapeutic target due to its role in pro-inflammatory cytokines production in AD and PD (Fellner et al., 2013; Heneka et al., 2013; Burguillos et al., 2015). Recently, Slusarczyk et al. (2018), demonstrate the role of the antidepressant compound Tianeptine, in the inhibition of NLRP3 and TLR4 related pathways, reducing microglial activation. Similar to this compound, Resveratrol (a phenol compound) treatment also prevented the pro-inflammatory effect of fibrillar Aβ on macrophages by inhibiting STATs and NFkB related pathways, affecting to TNF-α or IL-6 levels.

However, the reduction of the pro-inflammatory phenotype was not connected to an increase of a more anti-inflammatory phenotype. Recently, Chen and colleagues, use TGF-β1 to induce to promote the production of anti-inflammatory like phenotype that turns out to be protective in MPP+ (1-methyl-4-phenylpyridinium) PD-like rat model. TGF-β1 action takes place by blocking Smad3 inhibitor SIS3 and caspase-3/9 activity (Chen et al., 2017). Another strategy followed to reduce the impact of AD progression is the use of non-steroidal anti-inflammatory drugs (NSAID). The use of NSAID, as a long run strategy to counteract AD progression, remains controversial due to not fully conclusive data. However, in a recent meta-analysis performed by Zhang et al. (2018) (the meta-analysis includes 121 studies: 16 cohort studies with 236,022 participants and published between 1995 and 2016), the authors conclude that the use of NSAIDs is significantly associated with a reduction in the risk of developing AD.

To induce a shift in the microglial profile from anti- to pro-inflammatory state, would be another potential strategy to reduce the inflammatory response. For instance, IL-4 is an inflammatory molecule link to the “M2-like” phenotype in microglial cells. Recently, Kizil et al., demonstrated in 3D cultures amyloid-β42 reduces neural stem cell (NSC) plasticity by inducing kynurenic acid (KYNA) production. IL-4 restores NSC proliferative and neurogenic ability by suppressing the KYNA-producing enzyme Kynurenine aminotransferase (Papadimitriou et al., 2018). However, it has been reported that other cytokines traditionally considered anti-inflammatory cytokines, could play a detrimental role. For instance, Golde et al., demonstrate that IL-4 expression in the hippocampus leads to an increase in the amyloid deposition might be linked with reduced clearance mechanism (Chakrabarty et al., 2012). IL-10 is another anti-inflammatory cytokine shedding contradictory data on AD progression. For instance, Golde et al., demonstrated that IL-10 expression in APP/PS1 model resulted in increased expression in APP/impaired cognitive outcome in APP mice (Chakrabarty et al., 2015). Cytokines such as IL-12 have been also proposed as a therapeutic target. As previously comment, the lack of IL-12 reduces AD pathology in APP/PS1 mice, making this molecule an interesting therapeutic target. Indeed, Heppner’s lab demonstrated that the peripheral injection of p40 inhibitor (IL-12 subunit) is enough to reduce the amyloid burden (Vom Berg et al., 2012).

Over the last decade, it has been suggested the immunoproteasome as a valid target counteract the AD pathology in relation to microglial response and innate immune system. In 2012, Aso et al. (2012), showed in APP/PS1 mice the activation of the immunoproteasome over the disease progression. In 2013, Orre et al. (2013), confirmed the strong activation of the immunoproteasome in APP mice and using a specific immunoproteasome inhibitor they were able to reduce microglial activation *ex vivo*. Recently, in 2017, Prokop’s lab demonstrated that APP/PS1 mice lacking of immunoproteasome subunit 7 (LMP7) had a reduced inflammatory response (Wagner et al., 2017).

Another therapeutic approach considered to counteract the progression of neurodegenerative disease is the use of peripheral macrophages. Recently, Cronk et al. (2018), have demonstrated a unique microglial signature for brain-engrafted macrophages. These macrophages can carry out the regular microglial-like activity, being a potential tool to help microglial cells in the task of keeping brain homeostasis in disease brain context. However, other studies suggest those peripheral macrophages are not capable of carrying out microglial related tasks efficiently in the context of neurodegenerative diseases. For instance, Prokop et al. (2015), demonstrated that peripheral macrophages are insufficient to clear amyloid burden in APP/PS1 mice after depleting resident microglial cells. Furthermore, Neher’s lab, demonstrated that the monocyte repopulation for up to 6 months in APP/PS1 AD mouse model did not modify amyloid load, questioning the effectiveness of monocytes as a long-term therapy for AD (Varvel et al., 2015). Possibly, macrophage phagocytosis might not be as effective as microglia in amyloid beta up take (Fiala et al., 2005).

## Conclusion

Microglial state-of-art in neurodegenerative disorders shed light on the key role of the inflammatory response and microglial activation in the progression of neuronal disorders such as AD, PD or FTD. To understand the complex cellular interplay, we need to unravel the main mechanism controlling microglial activation. Turns out, microglia activation profile seems to be region and context dependent in brain disorders. As discussed over the review, gray or white matter content, aging, microglial content, re-organization or neuronal network and neuronal loss differ between regions and might have a clear impact in microglial performance in brain-related diseases, such as PD or AD.

Besides the role of the environment and cell interactions with microglial cells, another key factor driving specific microglial response is related to the expression and activation of different PRRs. The combination of different PRRs and the interaction with different ligands pictures the mosaic of potential microglial response. This heterogeneity represents a scientific challenge but also open the possibility to find new therapeutic targets to halt the progression of the different disorders.

The discrepancies on the role of the anti- and pro-inflammatory cytokines demonstrate the complexity of the microglial regulation in the brain. Therefore, further research is needed to unravel the frame windows where the modulation of either pro- or anti-inflammatory cytokines suits the different stages of the pathologies. For instance, at the beginning of the pathology some degree of inflammatory response might be needed, so that, the anti-inflammatory response might be reduced and *vice versa* in later stages of the pathology.

However, and despite of these differences, some similarities can be found in microglial activation linked to the inflammatory response elicited in the neurological disorders described in the review. The production and release of inflammatory mediators such as cytokines, ROS and RNS seem to be a common feature linked to microglial response, which is tightly related to neurological disorders where protein homeostasis is imbalanced, such as PD, AD and FTD. Besides the protein aggregation, microglial activation linked to neuronal dysfunction has been observed in the neurological disorders reviewed. Thus, protein aggregation and neuronal death might share some common mechanism governing microglial inflammatory response.

Despite of the similarities described, the region-specific differences and microglial heterogeneity of the response, needs to be unraveled in order to fully understand disease-dependent microglial activation mechanisms to find new therapeutic targets (Box 1).

BOX 1 **What can we do for better understanding of microglial phenotype in neuronal disorder?**

- Single-cell analysis of microglial cells isolated from different brain areas and diseases at different time points.
- To study the interplay between glial cells and neurons to identify new microglial regulatory mechanisms.
- To develop new mouse models where microglial related risk factors along with disease related mutations are together to study the role of microglial cells.
- Better understanding of inflammatory response dynamics, to differentiate different periods in the response.

## Author Contributions

All authors provided sections of text covering their area of expertise and participated in the proofreading and discussion. AB-S organized and supervised the review with the active collaboration of the rest of the authors. SB wrote about FTLD, IJ-F wrote the introduction and made the figure, YY and AP wrote about Alzheimer’s disease. MS and TD were involved in the writing process.

## Conflict of Interest Statement

The authors declare that the research was conducted in the absence of any commercial or financial relationships that could be construed as a potential conflict of interest.
